# Clinical and Biochemical Characterization of Specific *GUCY2D* Alleles Associated With a Rare Form of Night Blindness

**DOI:** 10.1167/iovs.66.6.22

**Published:** 2025-06-06

**Authors:** Rola Ba-Abbad, Igor V. Peshenko, Malena Daich Varela, Siying Lin, Gavin Arno, Omar A. Mahroo, Catherine Egan, Anthony G. Robson, Elena V. Olshevskaya, Alexander M. Dizhoor, Andrew R. Webster

**Affiliations:** 1 King Khaled Eye Specialist Hospital, Riyadh, Saudi Arabia; 2 Pennsylvania College of Optometry, Salus at Drexel University, Elkins Park, Pennsylvania, United States; 3 Moorfields Eye Hospital, London, United Kingdom; 4 UCL Institute of Ophthalmology, University College London, United Kingdom; 5 Division of Evolution, Infection and Genomics, School of Biological Sciences, Faculty of Biology, Medicine and Health, University of Manchester, Manchester, United Kingdom; 6 Manchester Centre for Genomic Medicine, Manchester Royal Eye Hospital, Manchester, United Kingdom; 7 Department of Ophthalmology, Saint Mary's Hospital, Manchester University NHS Foundation Trust, Manchester, United Kingdom

**Keywords:** *GUCY2D*, inherited retinal dystrophy, night blindness

## Abstract

**Purpose:**

To clinically and biochemically characterize a rare autosomal recessive rod-cone dysfunction, with electroretinographic similarities to some forms of stationary night blindness (SNB), associated with biallelic variants in *GUCY2D*.

**Methods:**

Six patients from five families with a history of longstanding night blindness, no fundus features suggestive of retinitis pigmentosa, and an unusual electroretinographic phenotype were ascertained. Clinical examination and genotyping were performed. Selected *GUCY2D* variants were tested for binding to and activation by guanylate cyclase-activating proteins (GCAPs) in HEK293 cells.

**Results:**

The visual acuity was normal or moderately reduced (20/20–20/60) with three patients having a tritan defect on color vision testing. Retinal imaging showed central macular hypopigmentation with temporal vascular attenuation. Rod photoreceptor-mediated electroretinogram (ERG) components were undetectable or severely reduced in all but one case, and cone-mediated responses were variable. A high degree of ERG stability was demonstrated in three cases. Molecular analyses revealed biallelic variants of *GUCY2D* in all patients, four of which are clinically and biochemically characterized for the first time, to our knowledge. These allelic variants encoded retinal guanylyl/guanylate cyclase 1 (RetGC1) mutants whose enzymatic activities were significantly diminished due to drastically reduced affinity of RetGC1 for GCAPs.

**Conclusions:**

The apparent lack of retinal degeneration, clinical features, predominant and severe rod photoreceptor involvement, and relatively high degree of ERG stability are similar to rare forms of SNB. Biallelic disease-causing variants in *GUCY2D* are usually associated with Leber's congenital amaurosis (LCA); however, this study illustrates the phenotypic variability of *GUCY2D* retinopathies in association with variants not biochemically dissimilar to those causing LCA and highlights the complexity of RetGC1 regulation in rod and cone photoreceptor function.

Inherited retinal disorders (IRDs) are a genetically and phenotypically heterogeneous group of disorders that constitute a major cause of visual disability in the developed world.^1^^,^^2^ The most severe form of IRDs, Leber's congenital amaurosis (LCA), manifesting as severe visual loss from birth or early infancy, is associated with variants in 24 genes identified so far.^3^ Biallelic variants in the human retinal guanylyl/guanylate cyclase 1 (RetGC1) coding gene (*GUCY2D*, MIM: 600179) account for 10% to 20% of autosomal recessive LCA (LCA1, MIM: 204000). Specific variants in *GUCY2D* can cause autosomal dominant cone–rod dystrophy (CORD, MIM: 601777).^4^ More recently, a rare form of autosomal recessive congenital (stationary) night blindness has been associated with *GUCY2D* variants (SNB, MIM: 618555).^5^

*GUCY2D* encodes RetGC1, the main guanylate cyclase of the photoreceptors, which is an important element in modulating the photoresponse.^6^ Phototransduction starts from photoisomerization of the photopigment in the photoreceptor outer segments (OSs). This activates the photoreceptor G-protein transducin, which in turn activates the photoreceptor phosphodiesterase (PDE6), causing hydrolysis of cyclic guanosine monophosphate (cGMP) and closure of the cyclic nucleotide-gated (CNG) channels at the OS plasma membrane, ultimately resulting in reduction of intracellular cation influx and hyperpolarization of the photoreceptors.^6^ Low intracellular Ca^2+^ concentrations activate RetGC isozymes 1 and 2 through their interaction with Ca^2+^/Mg^2+^ binding proteins (guanylate cyclase-activating proteins [GCAPs]). Activated RetGC, predominantly RetGC1,^5^ replenishes cGMP, accelerates reopening of the CNG channels, and restores the partial depolarization state of the photoreceptor membrane potential.^7^

In vitro analysis of 45 recessive LCA-associated *GUCY2D* variants showed that they reduce or abolish cGMP synthesis, which would likely keep most of the CNG channels in the rods and cones closed.^8^ However, detailed phenotyping of LCA1 patients with detectable visual function showed cone-mediated vision to be more affected than that driven by the rods.^9^^,^^10^ In some patients with undetectable cone function, the rod-mediated function had an apparent lack of saturation even under high photopic levels, which was attributed to the absence of cone-mediated rod inhibition.^9^ Conversely, CORD-associated dominant *GUCY2D* variants are gain-of-function alleles exerting their action through shifting Ca^2+^-sensitivity, thus keeping the CNG channels open even under light-adapted conditions, eventually leading to photoreceptor degeneration.^8^

Recently, six patients with childhood-onset night blindness and rod more than cone dysfunction were reported to harbor biallelic *GUCY2D* variants.^5^^,^^11^ The associated genotypes did not cluster in a specific domain of RetGC1, despite the strong predilection to affect rod function. Because of the relatively mild nature of this disorder, the authors suggested that the associated missense changes are likely to be hypomorphic alleles.^5^

In this study, we determined the clinical and molecular features of a further six patients with predominant night blindness associated with *GUCY2D* alleles, identified through next-generation sequencing. This study also performed in vitro functional assessment of four of the identified *GUCY2D* variants and compared them to LCA1-associated alleles.

## Methods

This study adhered to the tenets of the Declaration of Helsinki and was approved by the Northwest Research and the Moorfields Eye Hospital ethics committees. Informed consent was obtained from all patients. The animal procedures for this study were approved by the Salus University Institutional Animal Care and Use Committee in accordance with the National Institutes of Health guidelines for animal use in research.

Patient 1 was evaluated as part of a wider project to explore the genetic causes of severe rod dysfunction in patients without (1) marked loss of cone function based on full-field electroretinogram (ERG), (2) typical features of retinitis pigmentosa, or (3) ON bipolar cell dysfunction.^12^ Patients 2 to 5 were subsequently identified from genome sequencing data in the 100,000 Genomes Project, based on similarities of phenotype and genotype.^13^ Finally, patient 6 was ascertained from the Oculome Panel test project, with revision of the phenotype from LCA to SNB through deep phenotyping.^14^

### Clinical Examination

All patients underwent full ophthalmic examination including best-corrected Snellen visual acuity (VA), color vision using the Ishihara pseudoisochromatic test or the Hardy–Rand–Ritter (HRR) color test. Retinal color or pseudocolor images were obtained using Topcon (Tokyo, Japan) or Optos (Dunfermline, United Kingdom) fundus imaging devices, respectively. Fundus autofluorescence (FAF) images were obtained with the Optos device, and spectral-domain optical coherence tomography (SD-OCT) scans were obtained using the SPECTRALIS HRA+OCT device (Heidelberg Engineering, Heidelberg, Germany). Full-field and pattern electroretinogram (PERG) testing was performed to incorporate the standards of the International Society for Clinical Electrophysiology of Vision (ISCEV).^15^^,^^16^ Photopic On–Off ERGs and S-cone ERGs were also performed.^17^^,^^18^

### Molecular Genetics

Leukocyte DNA was extracted from all participants. Patient 1 underwent whole exome sequencing (WES) as described previously.^12^ The phase of variants was determined by Sanger sequencing of parental DNA. Patients 2 to 5 and their parents underwent whole genome sequencing through the NHS-England Genomic Medical Service (GMS) as described previously.^13^ Patient 6 underwent, as part of the GMS, custom clinical exome enrichment and sequencing as described previously^14^; parental testing was not performed.

### Biochemical Methods

#### *GUCY2D*  Mutagenesis 

*GUCY2D* mutations were introduced in human RetGC1 cDNA in a modified pRc/CMV-based construct^19^^,^^20^ using the “splicing by overlap extension” technique^21^ utilizing the following pairs of overlapping mutation-coding primers:

• p.(Tyr555Ser)—5′-AACATTGGTGTCTcTGAGGGAGACAGGGTTTG and 5′-CAAACCCTGTCTCCCTCAgAGACACCAATGTT
• p.(Arg624Gln)—5′-CAAGCTCCAGGAGgTCCGGCATGAG and 5′- CTCATGCCGGAcCTCCTGGAGCTTG
• p.(Leu587Val)—5′-TCAGAGCACTGCACGCagGGCTCTCTTCAG and 5′- CTGAAGAGAGCCctGCGTGCAGTGCTCTGA
• p.(Gly653Glu)—5′- GCTGGACCTTATCAAGGaAATAAGGTACCTGC and 5′-GCAGGTACCTTATTtCCTTGATAAGGTCCAGC

The fragments for each mutant were PCR-amplified using a Thermo Scientific Phusion Flash DNA polymerase (Thermo Fisher Scientific, Waltham, MA, USA). They were then inserted into the AgeI/DraIII restriction endonucleases sites of the modified mOrange–*GUCY2D* cDNA construct,^20^ and then a fragment coding for the extracellular domain of the cyclase amplified from wild-type *GUCY2D* cDNA was inserted into the HindIII/AgeI sites of each construct, using the 5′-ACCCAAGCTTGATATCGAATTCCGGCCCA and 5′-TGAAGTAACCGGTGCCTCACATAATGG primers, to replace the mOrange tag with the full-size extracellular domain of *GUCY2D*/RetGC1 in the expressed protein. All constructs used in the study were verified by Sanger sequencing on both strands.

#### *GUCY2D* Expression

Wild-type and mutant *GUCY2D* gene products were expressed in human embryonic kidney 293 (HEK293) cells from a modified Invitrogen (San Diego, CA, USA) pRc/CMV vector using the calcium phosphate DNA precipitation transfection protocol, and the membrane fraction containing the recombinant RetGC1 was isolated as detailed previously.^22^ The activity of the cyclase was assayed using [α-^32^P] guanosine-5'-triphosphate (GTP) (PerkinElmer, Waltham, MA, USA) as a substrate, and the [^32^P]cGMP product was quantified using thin-layer chromatography (TLC) as described previously.^22^ Briefly, the assay mixture (25 µL) incubated at 30°C contained 30-mM 3-(*N*-morpholino)propanesulfonic acid (MOPS)–potassium hydroxide (KOH) (pH 7.2), 60-mM potassium chloride (KCl), 4-mM sodium chloride (NaCl), 1-mM dithiothreitol (DTT), 2-mM Ca^2+^/EGTA buffer, 1-mM or 6-mM free Mg^2+^ as indicated in the text, 0.3-mM adenosine triphosphate (ATP), 4-mM cGMP, 1-mM GTP, and 1 µCi of [α-^32^P]GTP. The resultant [^32^P]cGMP product was analyzed by TLC using fluorescently backed polyethyleneimine cellulose plates (Merck, Rahway, NJ, USA) developed in 0.2-M lithium chloride (LiCl) and eluted with 2-M LiCl, followed by liquid scintillation counting of the radioactivity.

#### GCAP Expression

Human GCAP1 (Ser6) was expressed from a Novagen/Calbiochem (Darmstadt, Germany) pET-11d vector in a BLR(DE3) *Escherichia* *coli* strain harboring yeast *N*-myristoyl transferase and purified by hydrophobic interaction chromatography and size-exclusion chromatography to ∼95% purity by SDS-PAGE as previously described.^23^ For co-transfection experiments in HEK293 cells, bovine GCAP1 was expressed tagged at the C-terminus with SuperGlo (Clontech, Mountain View, CA, USA) enhanced green fluorescent protein (GFP) using pQBIfN3 vector (Clontech) as previously described.^23^^,^^24^ Human GCAP2 cDNA was PCR-amplified from a human retinal cDNA library (StrataGene, La Jolla, CA, USA), inserted into the NcoI/BamHI sites of pET-11d, and, after verification by sequencing, expressed in *E. coli* and purified as previously described for bovine and mouse GCAP2.^25^^,^^26^ Human GCAP3 cDNA, main splice variant,^27^ was chemically synthesized (Genewiz, South Plainfield, NJ, USA), inserted into the NcoI/BamHI sites of the pET-11d vector, and expressed in *E. coli* following protocol for the isolation of myristoylated GCAP2. The purity of the recombinant GCAP2 and GCAP3 expressed in *E. coli* was >90% by SDS-PAGE/Coomassie Blue stain.

#### Ca^2+^/EGTA Buffers

Ca^2+^/EGTA buffers maintaining defined free Ca^2+^ and Mg^2+^ concentrations in the RetGC1 assay were calculated, prepared, and verified by fluorescent Ca^2+^ indicator dyes as previously described in detail.^28^

#### Co-Transfection Experiments

The mOrange-tagged RetGC1 constructs were co-expressed with the SuperGlo GFP-tagged bGCAP1 in HEK293 cells at the cyclase-to-GCAP1 plasmid ratio of ∼100:1 as described in detail previously,^20^ using ∼3 µL/µg FuGENE HD Transfection Reagent (Promega, Madison, WI, USA). Confocal images were taken utilizing an FV1000 confocal laser scanning biological microscope (OLYMPUS, Tokyo, Japan) using 543-nm and 488-nm excitation for the red and the green fluorochromes, respectively, collected in a sequential mode. No changes to the original images were made except for minor gamma correction applied to the whole image. Fluorescence intensities were compared by scanning non-corrected images.

#### Retinal Tissue

Mouse retinae were harvested from dark-adapted GCAPs^–/–^ mice^29^ under infrared illumination, and the tissue was processed for guanylyl cyclase assay as previously described.^26^^,^^30^ Assays using dark-adapted mouse retinae were conducted under infrared illumination using infrared goggles following a procedure previously described^31^; the reaction mixture in the assay was supplemented with creatine kinase and phosphocreatine.^32^

## Results

### Clinical and Electrophysiological Features

**Figure 1.**
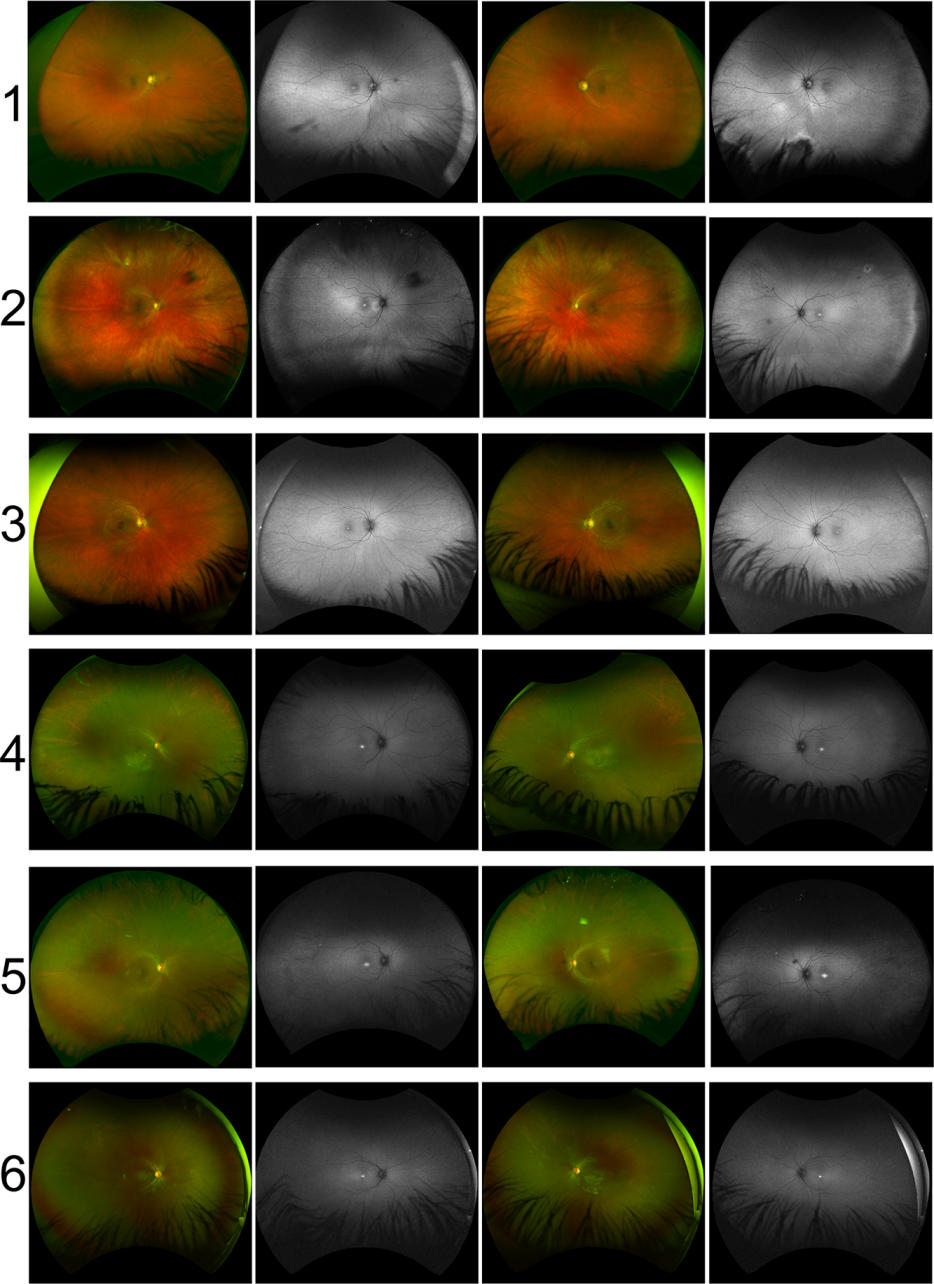
Ultra-widefield retinal pseudocolor and autofluorescence (AF) images for patients 1 to 6. Except for patient 3, all patients had temporal retinal vascular attenuation. The left fundus image of patient 1 showed an inferonasal alteration, better delineated on AF as a patch of reduced AF outlined by an increased signal. All patients had a hyperautofluorescent spot at the macular center.

**Figure 2.**
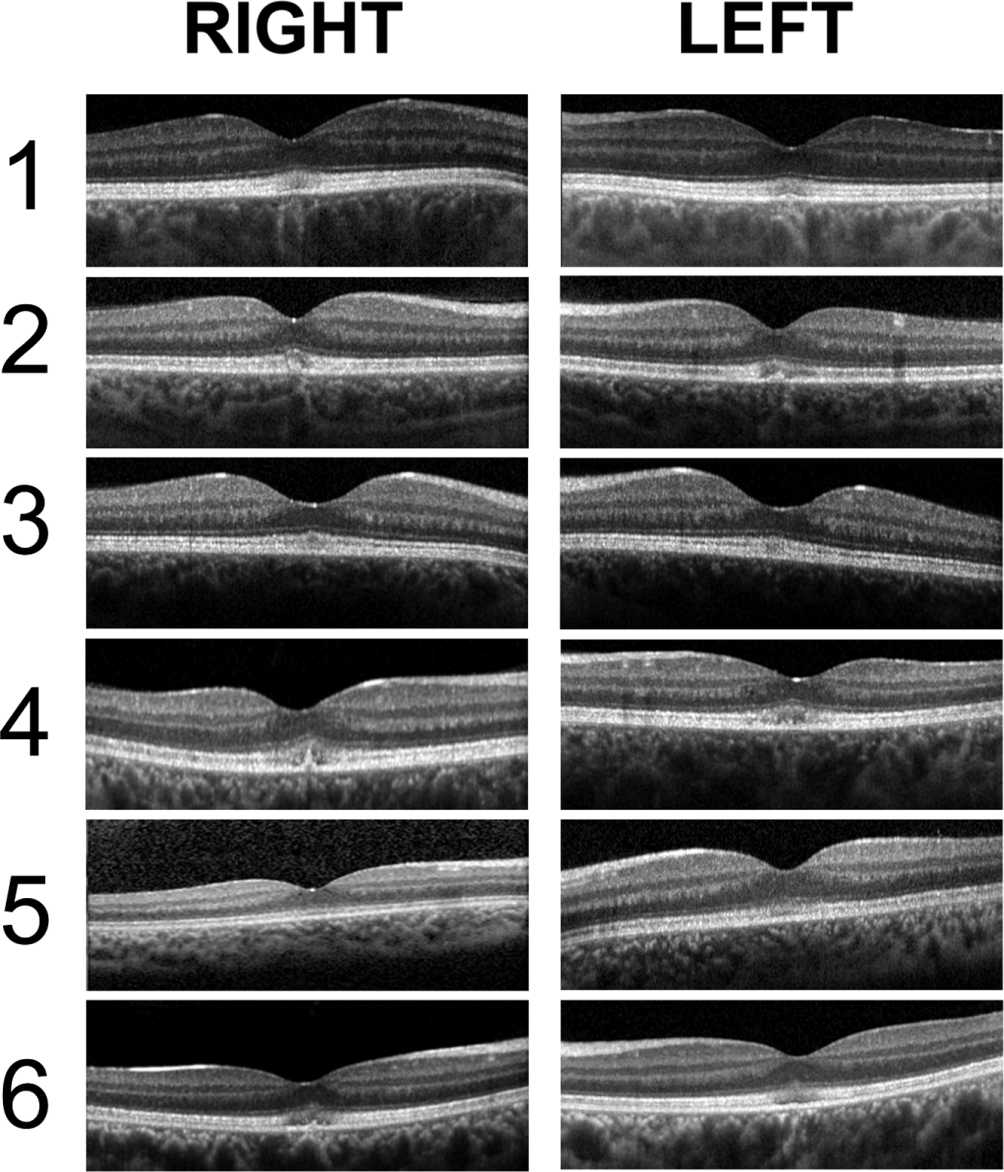
OCT scan of the right and left eyes of patients 1 to 6. All patients had variable degrees of disruption of the foveal ellipsoid–interdigitation zones. Patients 2 and 4 had a small vitelliform-like lesion at the fovea.

**Figure 3.**
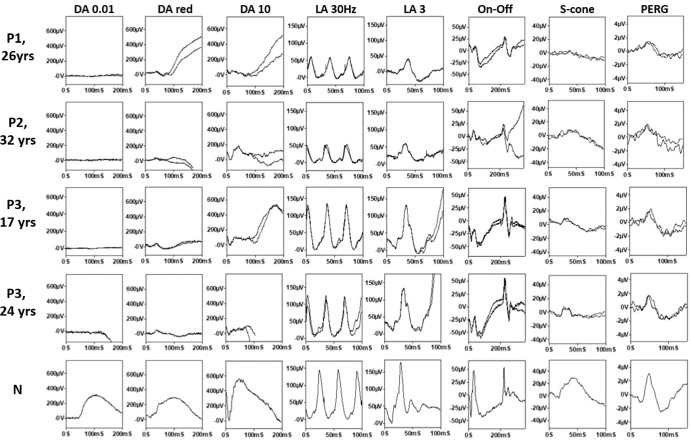
ISCEV standard full-field ERGs. On-Off ERGs, S-cone ERGs, and PERG from one eye each of patients 1 (P1, 26 years), 2 (P2, 32 years), 3 (P3 at 17 years and 24 years, respectively), and representative normal (N) examples for comparison. Standard ERGs are to xenon flashes of light, and all responses were recorded using gold foil corneal electrodes. Recordings showed a high degree of interocular symmetry and are shown from one eye only, with patient traces superimposed to demonstrate reproducibility.

**Figure 4.**
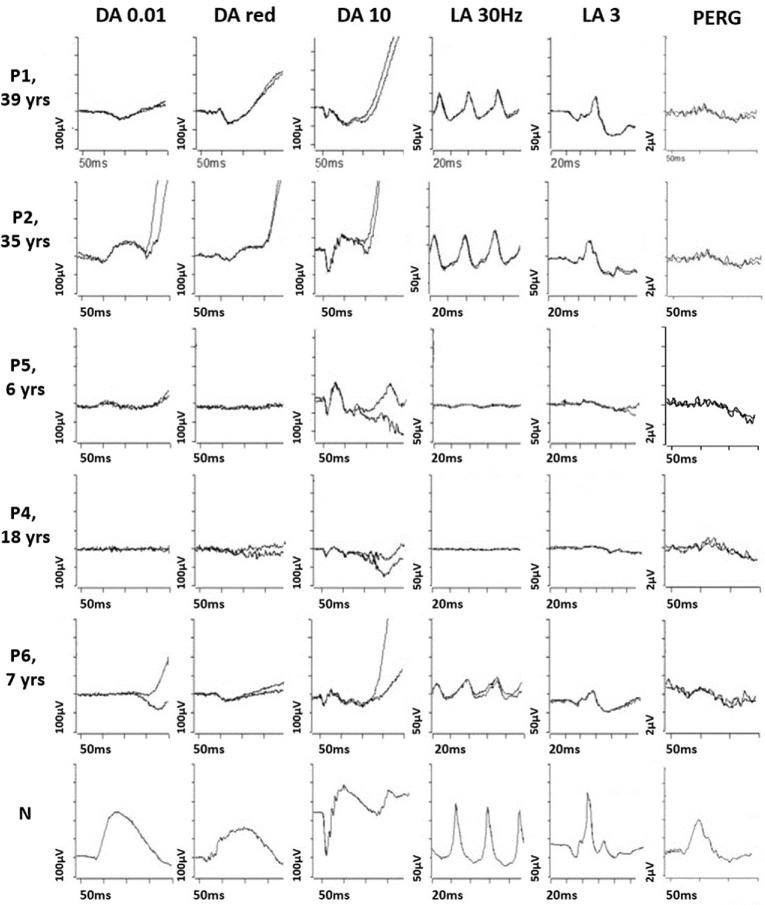
ISCEV standard full-field ERGs and PERG from one eye each of patients 1 (P1, 39 years), 2 (P2, 35 years), 4 (P4, 6 years), 5 (P5, 6 years), 6 (P6, 8 years), and representative normal (N) examples for comparison. Standard ERGs were recorded with LED flashes of white light and were recorded with gold foil corneal electrodes, although patient 4 (S4) was tested with silver thread electrodes. Recordings showed a high degree of interocular symmetry and are shown from one eye only, with patient traces superimposed to demonstrate reproducibility.

The clinical and ERG characteristics of this cohort are summarized in Table 1. The age at the last clinical assessment ranged between 13 and 42 years. All patients had childhood-onset nyctalopia with normal to moderately reduced VA (20/20–20/60). When available, color vision screening showed normal red–green discrimination and a tritan defect. Anterior segment examination was unremarkable in all patients, and there was no nystagmus. Except for patient 3, all patients had temporal retinal vascular attenuation (Fig. 1), and some had central macular hypopigmentation (Supplementary Fig. S1). The FAF images (Fig. 1) showed central macular hyperautofluorescence corresponding to variable degrees of ellipsoid zone disruption on OCT (Fig. 2). All patients had undetectable rod-specific dark-adapted (DA) 0.01 ERGs with cone system dysfunction (Figs. 3, 4). The clinical histories and specific retinal findings are given below.

**Table 1.** Demographic, Clinical, and Electroretinographic Features of Rod–Cone Dysfunction in Six Patients With Biallelic Variants in *GUCY2D*

Patient	Assigned Sex at Birth	Onset of Symptoms (y)	Last Assessment (Y)	Snellen VA	Color Vision	Fundus Features	Fundus Autofluorescence	Macular OCT	DA 0.01	DA 10.0	LA 3.0 30-Hz
1	Male	11	42	20/30; 20/20	Tritan defect	Mild temporal vascular attenuation; blunted foveal reflex with hypopigmentation at the macular center; mild left outer retinal alterations at the inferonasal quadrant	Hyperautofluorescence at the macular center. Left patch of hypoautofluorescence in the inferonasal quadrant with a posterior edge of hyperautofluorescence	EZ disruption and thickening of the interdigitation zone	UD	Subnormal a-wave; subnormal b:a ratio	Profound delay; normal amplitudes
2	Female	Lifelong	35	20/30 OU	Tritan defect	Mild temporal vascular attenuation; blunted foveal reflex with hypopigmentation at the macular center	Hyperautofluorescence at the macular center	Vitelliform lesions	Subnormal	Subnormal a-wave; b-wave had short peak time; normal b:a ratio	Mild delay; subnormal amplitudes
3	Female	10	18	20/20 OU	Normal red–green color discrimination	Hypopigmentation at the macular center	Hyperautofluorescence at the macular center	EZ disruption and mild thickening at the fovea	UD	Subnormal a-wave; b-wave had short peak time; subnormal b:a ratio	Profound delay; normal amplitudes
4	Female	Longstanding	18	20/60 OU	NA	Temporal vascular attenuation	Hyperautofluorescence at the macular center	Vitelliform lesions	UD	Subnormal a-wave; b-wave had short peak time; normal b:a ratio	Profound delay; severely reduced amplitudes
5	Female	Longstanding	13	20/60 OU	NA	Temporal vascular attenuation	Hyperautofluorescence at the macular center	Hypo-reflective EZ at the foveal center	UD	Subnormal a- and b- waves of unusually short peak time	UD
6	Female	Longstanding	16	20/25; 20/30	Tritan defect	Temporal vascular attenuation with hypopigmentation at the macular center	Hyperautofluorescence at the macular center	Vitelliform lesions	UD	Subnormal a- and b- waves of unusually short peak time	Profound delay; subnormal amplitudes

EZ, ellipsoid zone; NA, not available; OU, both eyes; UD, undetectable.

#### Patient 1 (GC25541)

A 27-year-old man of European ancestry had reduced night vision noticed at the age of 11 years, resulting in a diagnosis of retinitis pigmentosa. He struggled under flood illumination and dusk-like light levels and had difficulty distinguishing between shades of green and blue. He had difficulty tracking small fast-moving objects such as cricket or golf balls. He was otherwise in good health. On his most recent examination (at age 42), color contrast thresholds were normal along the protan and deutan axes but showed elevation along the tritan axis.^33^ The retinal images and features are given in Figure 1, Supplementary Figure S1, and Table 1. Notably, there was a small region of outer retinal alteration in the inferonasal midperiphery of the left eye.

**Figure 5.**
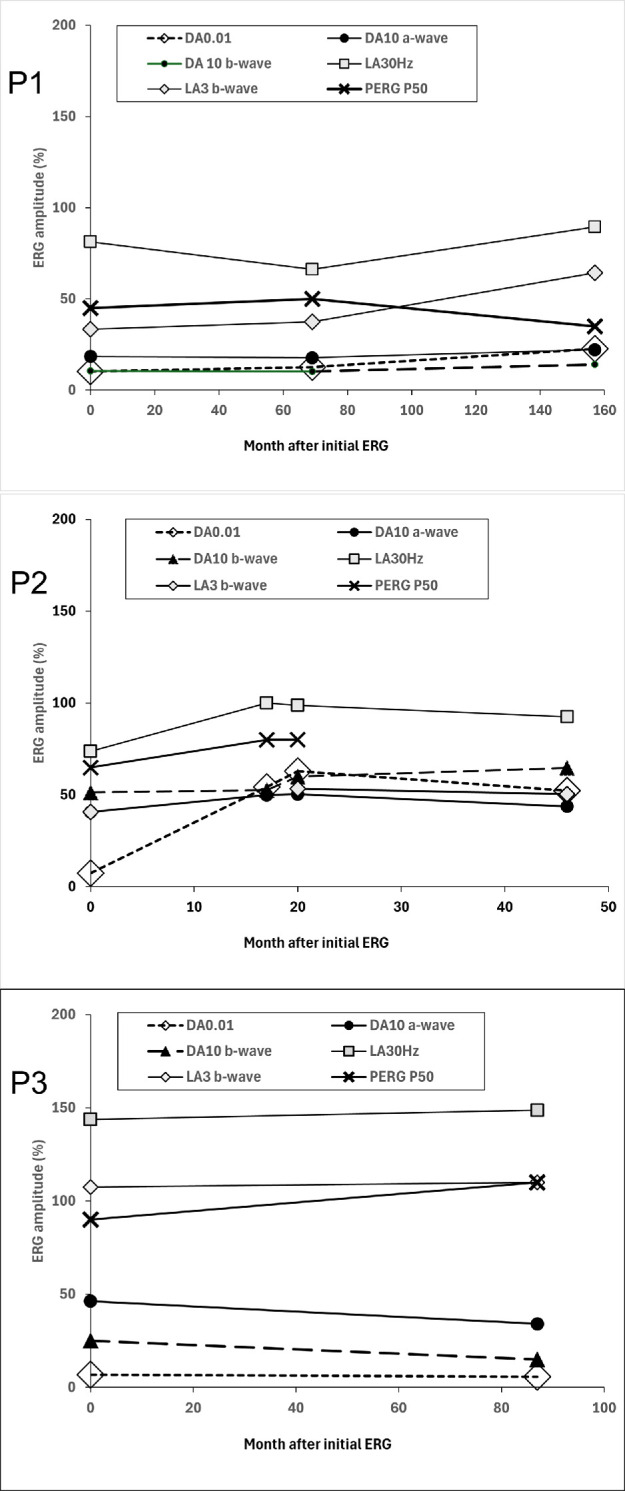
Graphs showing stability of the main full-field ERG and PERG P50 component amplitudes in patients 1 (P1), 2 (P2), and 3 (P3). All amplitudes are plotted as a percentage of the age-matched lower 5th percentile to allow direct comparison of DA and LA responses and recordings to xenon and LED-based stimuli (LED stimuli used to obtain final follow-up ERGs in patients 1 and 2). Note that baseline DA 0.01 ERGs in P2 were undetectable, but likely partly due to technical factors (responses at the three follow-up visits were consistently relatively preserved in comparison).

Dark-adapted and light-adapted (LA) full-field ERGs were performed at the age of 26 years (Fig. 3). The dim flash rod (DA 0.01) ERG was almost undetectable. The strong flash (DA 10) ERG showed a markedly subnormal a-wave and subnormal b:a ratio, with a b-wave of short peak time, similar to the DA red flash ERG waveform and consistent with a predominant contribution from the DA cone system. LA flicker (30 Hz) ERG was severely delayed (by 14 ms) with relatively mild amplitude reduction, and single flash cone (LA 3) ERGs were also delayed and of subnormal amplitude. Photopic On-Off ERG revealed greater delay in the b- than d-waves, and a subnormal b:a ratio. The S-cone ERG was undetectable. The PERG P50 was reduced, consistent with mild macular dysfunction (Fig. 3). Repeat testing (at the age of 32 years) after prolonged (overnight) dark adaptation of one eye had no effect on ERGs. Further ERG and PERG were performed (age 39 years), revealing no evidence of worsening retinal or macular function over more than 13 years (Figs. 4, 5).

#### Patient 2 (GC22985)

A 35-year-old woman of European ancestry complained of a mild decrease in the VA of both eyes, from 20/15 to 20/30. She had lifelong nyctalopia and difficulty in dim light. Upon direct questioning, after dark-adapting for 45 minutes, the patient noted that she was able to see the surrounding objects. The medical history was significant for kyphosis, bronchial asthma, and vitamin D deficiency for which she was receiving supplements, with no history of malabsorption. Her serum vitamin A level was reported to be normal. There was no family history of night blindness. Color vision testing showed normal red–green color discrimination on the HRR test, with a mild defect in the tritan axis. The retinal changes depicted in Figure 1 remained unchanged over a 4-year follow-up period.

Baseline DA 10 ERG at the age of 32 years showed subnormal a-waves and a b-wave of short peak time, of similar timing to the preserved DA red flash ERG x-wave, in keeping with a predominant DA cone system contribution (Fig. 3). LA 30-Hz ERG was mildly delayed and subnormal, and the LA 3 ERG was subnormal. On-Off ERG b- and d-waves were of normal timing, but the b:a ratio was subnormal. S-cone ERGs were just within the reference range. PERG P50 was reduced and stable over a 17-month period, consistent with macular dysfunction. Overnight dark adaptation of one eye had no effect on ERGs. Further ERGs performed nearly 4 years after initial testing revealed no evidence of worsening retinal function (Figs. 4, 5).

#### Patient 3 (GC18451)

A 17-year-old Caucasian British woman had long-standing difficulty with night vision, first noticed at the age of 10 years. The retinal features depicted in Figure 1 were stable over 13 years of follow-up. At the age of 17 years, DA 0.01 ERGs were residual, and responses to stronger flashes (DA 10 ERGs) had a subnormal a-wave, subnormal b:a ratio and b-wave peak of short peak time, similar to that of the DA red flash ERG x-wave (Fig. 3). LA 30-Hz and LA 3 ERGs were markedly delayed and of normal amplitude. On-Off ERG b- and d-waves were delayed and there was a low b:a ratio bilaterally. The S-cone ERG components were subnormal bilaterally. pERG P50, performed on the left eye at the final visit, was normal, revealing no evidence of macular dysfunction. Overnight dark adaptation of one eye failed to reveal a significant interocular ERG asymmetry. Repeat testing more than 7 years after baseline ERGs (at age of 24 years) revealed no evidence of worsening rod or cone system function (Figs. 2D, 4C).

#### Patients 4 and 5 (GC31207)

Patients 4 and 5 were female siblings of Southeast Asian ancestry, 18 years and 13 years old, respectively. Patient 4 had longstanding difficulties with seeing in the dark, with more recent problems with central vision. She had mild vitiligo but was otherwise fit and well. The retinal features (Fig. 1) are described in Table 1. When patient 4 was 18 years old, ERGs were performed with corneal electrodes according to the ISCEV standard. The DA 0.01 ERGs were undetectable, and there was marked DA 10 ERG a-wave reduction; the b:a ratio was normal, but b-waves had a short peak time, similar to that of the LA 3 ERGs (Fig. 4). The DA red flash ERG was undetectable. LA 30-Hz ERGs were delayed and severely reduced, and LA 3 ERGs were severely subnormal. The S-cone ERGs were detectable bilaterally. PERG P50 was delayed and markedly subnormal (P50 peak time 65 ms, amplitude 0.5 µV). The findings were consistent with severe generalized rod and cone dysfunction with marked macular involvement.

Patient 5 was a younger sibling of patient 4 with similar symptoms of poor vision under dim illumination. The clinical features are given in Table 1 and shown in Figure 1. At the age of 6 years, her ERG phenotype was very similar to that seen in her older sibling, showing evidence of severe generalized rod and cone photoreceptor dysfunction with delayed and markedly subnormal PERGs, consistent with severe macular involvement (Fig. 4D).

#### Patient 6 (GC31196)

Patient 6 was a 16-year-old Sri Lankan female who had childhood-onset difficulties with seeing under dim illumination and required additional support with navigation in the dark. She also had experienced difficulties with color discrimination since early childhood but denied photo-aversion. She had no other medical issues. Her retinal features are depicted in Figure 1 and summarized in Table 1. Color vision testing using HRR plates showed normal red–green color discrimination and a tritan defect. When she was 8 years old, DA 10 ERG a-waves showed marked reduction with b-wave peak times that were short and of similar timing to DA red flash ERG x-waves, suggesting a cone system origin. LA 30-Hz and LA 3 ERGs showed marked delay with moderately reduced amplitudes. PERG P50 was subnormal, in keeping with macular involvement.

### Molecular Characteristics

**Figure 6.**
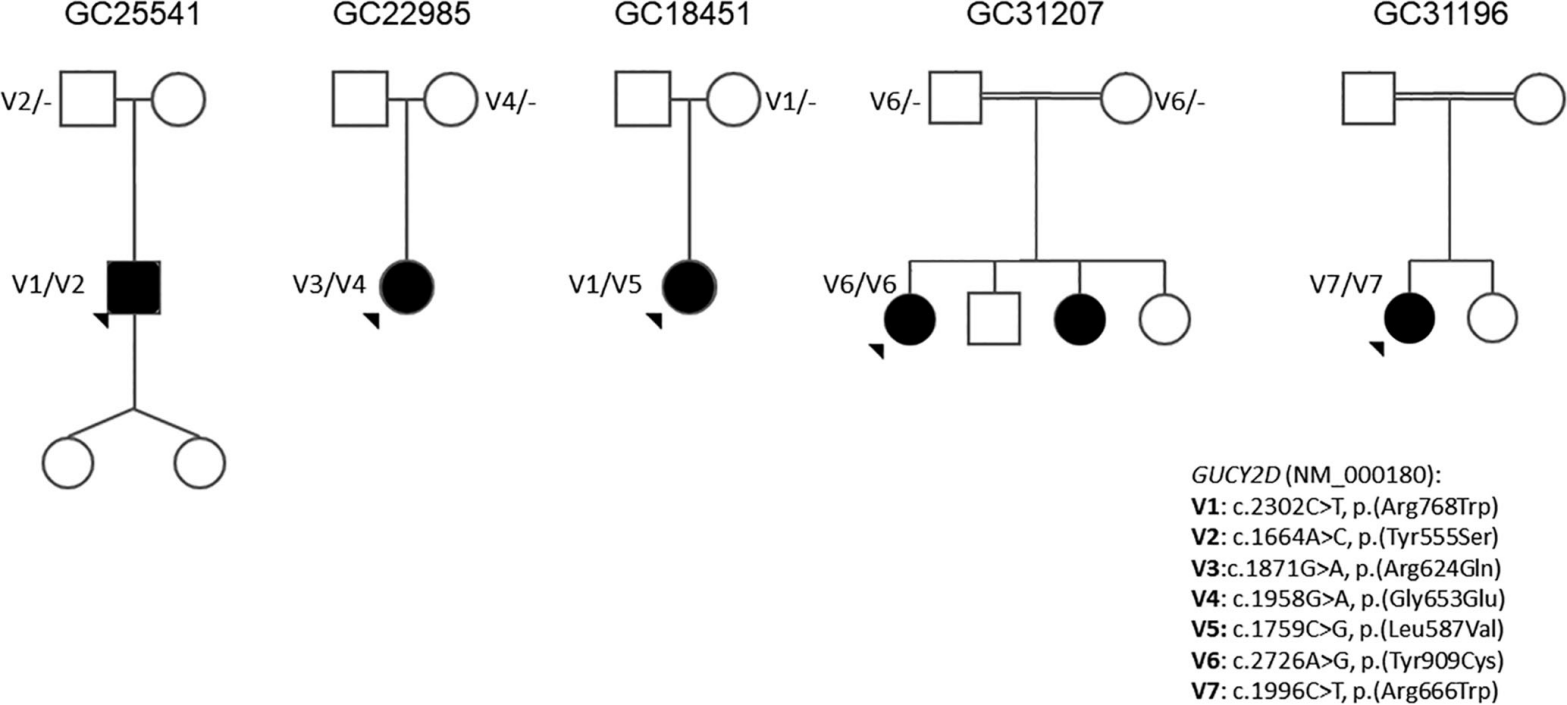
Pedigrees of all six patients. The affected family members are shown as solid symbols. Parental phasing is also shown.

The pedigrees and *GUCY2D* (NM_000180) variants detected by WES (patient 1) or whole-genome sequencing (patients 2–5) are shown in Figure 6 and summarized in Table 2. Patients 1 to 3 were compound heterozygotes for missense changes, and patients 4 to 6 were homozygotes for missense changes. Patients 1 and 3 had the known *GUCY2D* LCA variant: c.2302C>T, p.(Arg768Trp), likely in trans with previously unpublished missense changes. The variants in this cohort are in the following *GUCY2D* domains: juxtamembrane domain, kinase homology domain, and cyclase catalytic domain (Table 3). Except for the p.(Arg768Trp) pathogenic allele, all of the other variants have very low allele frequencies in the Genome Aggregation Database (gnomAD) and are classified as variants of uncertain significance according to American College of Medical Genetics and Genomics (ACMG) criteria (Table 3).

**Table 2.** Combinations of *GUCY2D* (NM_000180) Variants Associated With Stationary Night Blindness in This Case Series and Previous Studies

ID	Variant Combination	Ref.
1	c.2302C>T, p.(Arg768Trp); c.1664A>C, p.(Tyr555Ser)	This report
2	c.1871G>A, p.(Arg624Gln); c.1958G>A, p.(Gly653Glu)	This report
3	c.1759C>G, p.(Leu587Val); c.2302C>T, p.(Arg768Trp)	This report
4, 5	c.2726A>G, p.(Tyr909Cys) homozygous	This report
6	c.1996C>T, p.(Arg666Trp) homozygous	Patel et al.^14^
7	c.2302C>T, p.(Arg768Trp); c.2731C>T, p.(Leu911Phe)	Stunkel et al.^5^
8	c.247C>T, p.(Arg83Cys); c.1633C>T, p.(Gln545Ter)	Stunkel et al.^5^
9	c.1996C>T, p.(Arg666Trp); c.2945del, p.(Gly982ValfsTer39)	Stunkel et al.^5^
10	c.2302C>T, p.(Arg768Trp); c.2281C>T, p.(Arg761Trp)	Stunkel et al.^5^
11	c.1924T>G, p.(p.Phe642Val); c.2281C>T, p.(Arg761Trp)	Rodilla et al.^11^

**Table 3.** Characteristics of the *GUCY2D* (NM_000180) Variants Associated With Stationary Night Blindness in This Case Series and Previous Studies

Variant	Protein Domain	Allele Frequency (gnomAD)	ClinVar Accession Number	ACMG Classification (Franklin)	Known LCA1 Allele	Biochemical Properties	Ref.
c.247C>T, p.(Arg83Cys)	ECD	0.000001	VCV000689385.1	Variant of uncertain significance	No	NA	Stunkel et al.^5^
c.1664A>C, p.(Tyr555Ser)	JMD	0.000001	NA	Variant of uncertain significance	No	Not activated by GCAP	This report
c.1759C>G, p.(Leu587Val)	JMD	0.00007	VCV002194245.1	Variant of uncertain significance	No	Not activated by GCAP	This report
c.1871G>A, p.(Arg624Gln)	KHD	0.00001	VCV000957183.6	Variant of uncertain significance	No	Not activated by GCAP; severely reduced Ca^2+^ sensitivity	This report
c.1924T>G, p.(Phe642Val)	KHD	NA	VCV000974637.1	Likely pathogenic	No	NA	Rodilla et al.^11^
c.1958G>A, p.(Gly653Glu)	KHD	0.000007	VCV001390185.4	Variant of uncertain significance	No	Not activated by GCAP	This report
c.1996C>T, p.(Arg666Trp)	KHD	0.00002	VCV000860894.9	Variant of uncertain significance	No	Affects binding of GCAP1, but not RD3, with RetGC1; low-level of activation by GCAP1	Stunkel et al.^5^
c.2281C>T, p.(Arg761Trp)	KHD	0.00002	VCV000854601.6	Variant of uncertain significance	No	Affects binding of GCAP1 and RD3 with RetGC1; not activated by GCAP	Stunkel et al.^5^
c.2302C>T, p.(Arg768Trp);	KHD	0.0003 (one homozygote)	VCV000098563.39	Pathogenic	Yes	Did not bind either GCAP1 or RD3; not activated by GCAP1	Stunkel et al.^5^
c.2726A>G, p.(Tyr909Cys)	CCD	0.000002	NA	Variant of uncertain significance	No	NA	This report
c.2731C>T, p.(Leu911Phe)	CCD	NA	VCV000689382.1	Variant of uncertain significance	No	RetGC1 retained the ability to bind GCAP1 in cyto but failed to effectively bind RD3; not activated by GCAP	Stunkel et al.^5^
c.1633C>T, p.(Gln545Ter)	JMD	0.000002	VCV000689384.1	Pathogenic	Yes	NA	Stunkel et al.^5^
c.2943delG, p.(Gly982ValfsTer39)	CCD	NA	NA	Pathogenic	Yes	RetGC1 retained the ability to bind GCAP1 in cyto but failed to effectively bind RD3; not activated by GCAP1	Stunkel et al.^5^

CCD, cyclase catalytic domain; ECD, extracellular domain; JMD, juxtamembrane domain; KHD, kinase homology domain.

### Functional Variant Testing

We conducted in vitro studies to test four variants seen in this cohort: p.(Tyr555Ser), p.(Leu587Val), p.(Arg624Gln), and p.(Gly653Glu). Functional testing of the variant p.(Arg768Trp) has been undertaken in the past,^34^^,^^35^ and it has been found to impair RetGC1 activation by GCAP1 and/or completely suppress its catalytic activity.

**Figure 7.**
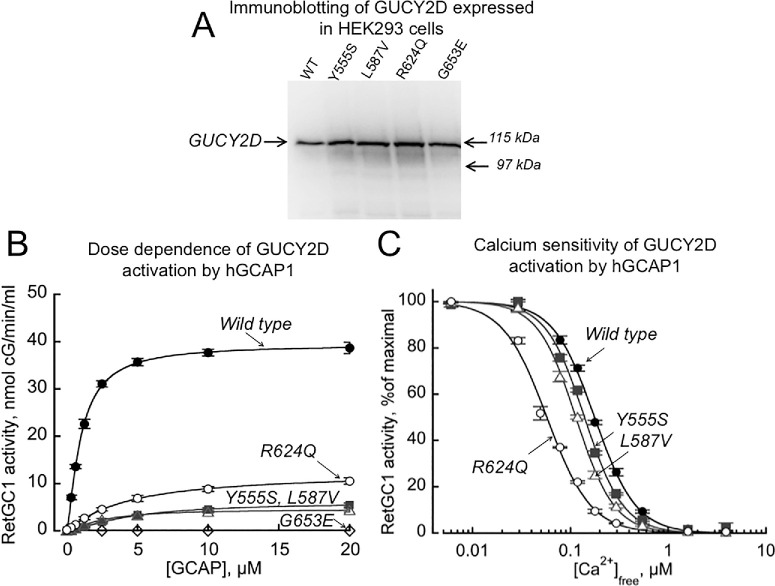
The effects of the *GUCY2D* variants on guanylyl cyclase activity and regulation by GCAP1. (**A**) Immunoblotting of the human *GUCY2D* expressed in HEK293 cells. The HEK293 cells were transfected with *GUCY2D* cDNA using calcium phosphate precipitation method and the membrane fractions from the transfected cultures were prepared as described in Methods. After the transfer from 7% SDS, the blot was probed by antibody against RetGC1 catalytic domain^53^ using a Thermo Scientific SuperSignal West Femto Chemiluminescence Substrate. The amount of the *GUCY2D* in subsequent biochemical analyses was normalized in all cases by the level of the chemiluminescence signal relative to the wild type. (**B**) Dose dependence of *GUCY2D* activation by GCAP1. The membrane fractions from HEK293 cells expressing wild-type, Y555S, L587V, R624Q, or G653E *GUCY2D* were reconstituted with purified recombinant human GCAP1 (hGCAP1) and assayed at saturating 10-mM MgCl_2_ in the presence of 2-mM EGTA. The data (mean ± SD) from three independent experiments were fitted (except for G653E, which had no detectable activity) using the function *A* = (*A*_max_)/(1 + ([GCAP]_1/2_/[GCAP])*^h^*), where *A* is the activity of the cyclase, *A*_max_ is the maximal activity at saturation with GCAP, [GCAP]_1/2_ is a GCAP concentration producing half-maximal stimulation, and *h* is a Hill coefficient. The respective *A*_max_ values (mean ± SD) from the fit for the wild-type, Y555S, L587V, and R624Q *GUCY2D* were 39.4 ± 0.4, 6.1 ± 0.35, 4.6 ± 0.2, and 12.4 ± 0.7 nmol/min/mL (activity in all mutants was different from the wild type (*P* <0.001, *t*-test). The respective [GCAP]_1/2_ values were 0.98 ± 0.03, 4.4 ± 0.43 (*P* = 0.005), 2.4 ± 0.2 (*P* = 0.005), and 4.1 ± 0.5 (*P* = 0.01). (**C**) The Ca^2+^ sensitivity of GCAP-stimulated activation for wild-type, Y555S, L587V, and R624Q *GUCY2D*. The cyclase activity in membranes containing recombinant *GUCY2D* was assayed in the presence of 14-µM GCAP1 and Ca^2+^/EGTA buffers, varying free Ca^2+^ concentrations in the presence of 0.9-mM free Mg^2+^ and normalized as a percentage of the maximal activity of each preparation. The data (mean ± SD, *n* = 3) were fitted assuming a Hill function, such that *A*% = 100/(1+([Ca]*_f_*/[Ca]_1/2_)*^h^*), were *A*% is the fractional activity of the cyclase, [Ca]*_f_* is a free Ca^2+^ concentration in the assay, [Ca]_1/2_ is a free Ca^2+^ concentration producing half-maximal inhibition of the cyclase activity, and *h* is a Hill coefficient. The [Ca]_1/2_ values for wild-type, Y555S, L587V, and R624Q *GUCY2D* were 176 ± 6, 139 ± 8 (*P* = 0.042), 114 ± 7 (*P* < 0.001), and 57 ± 7 (*P* < 0.001), respectively.

**Figure 8.**
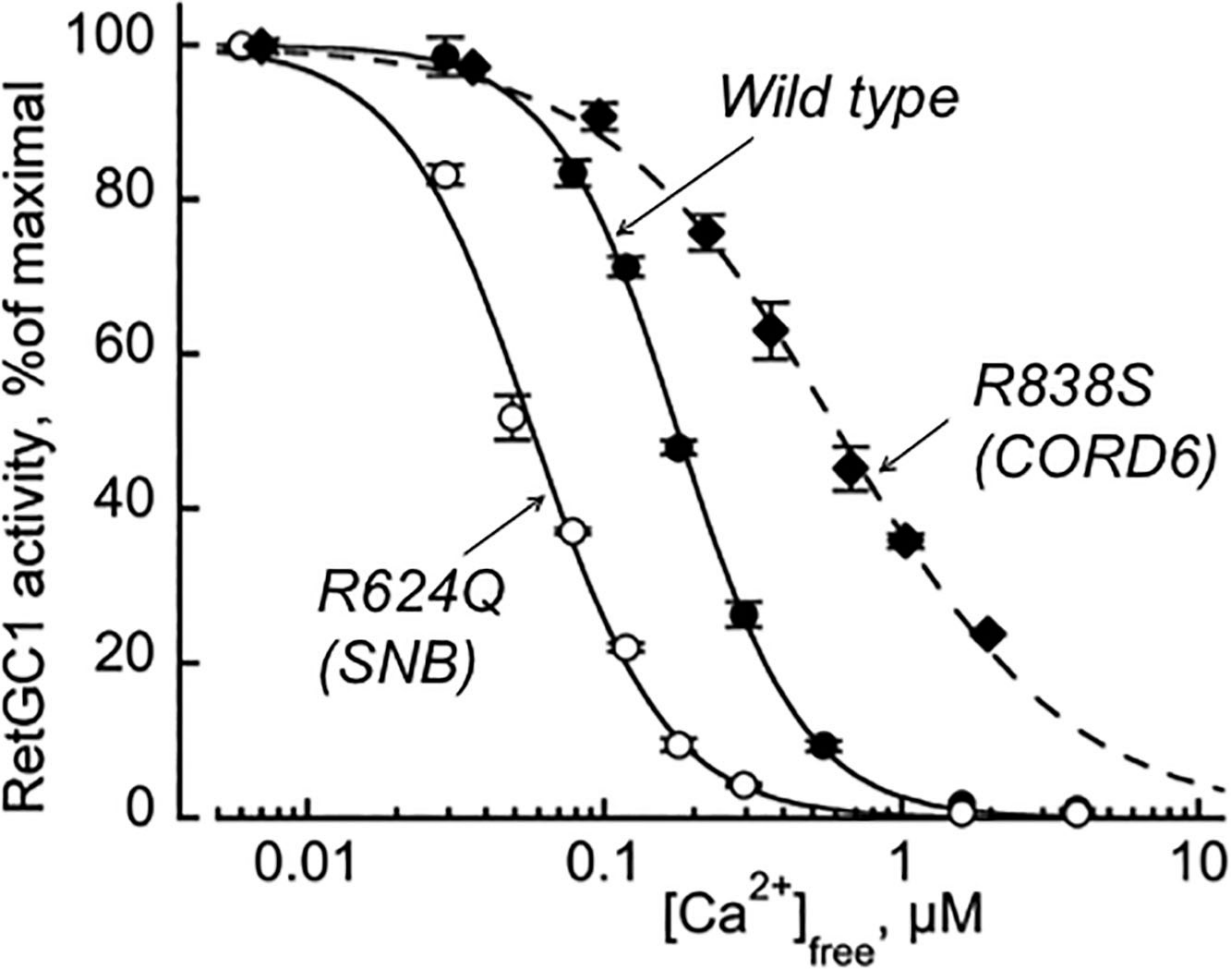
SNB and CORD6-linked mutations in *GUCY2D* alter the Ca^2^^+^ sensitivity of cyclase regulation in the opposite manner. Ca^2^^+^ sensitivity of the wild-type, R624Q, and R838S *GUCY2D* (the R838S data are from Dizhoor et al.^54^).

**Figure 9.**
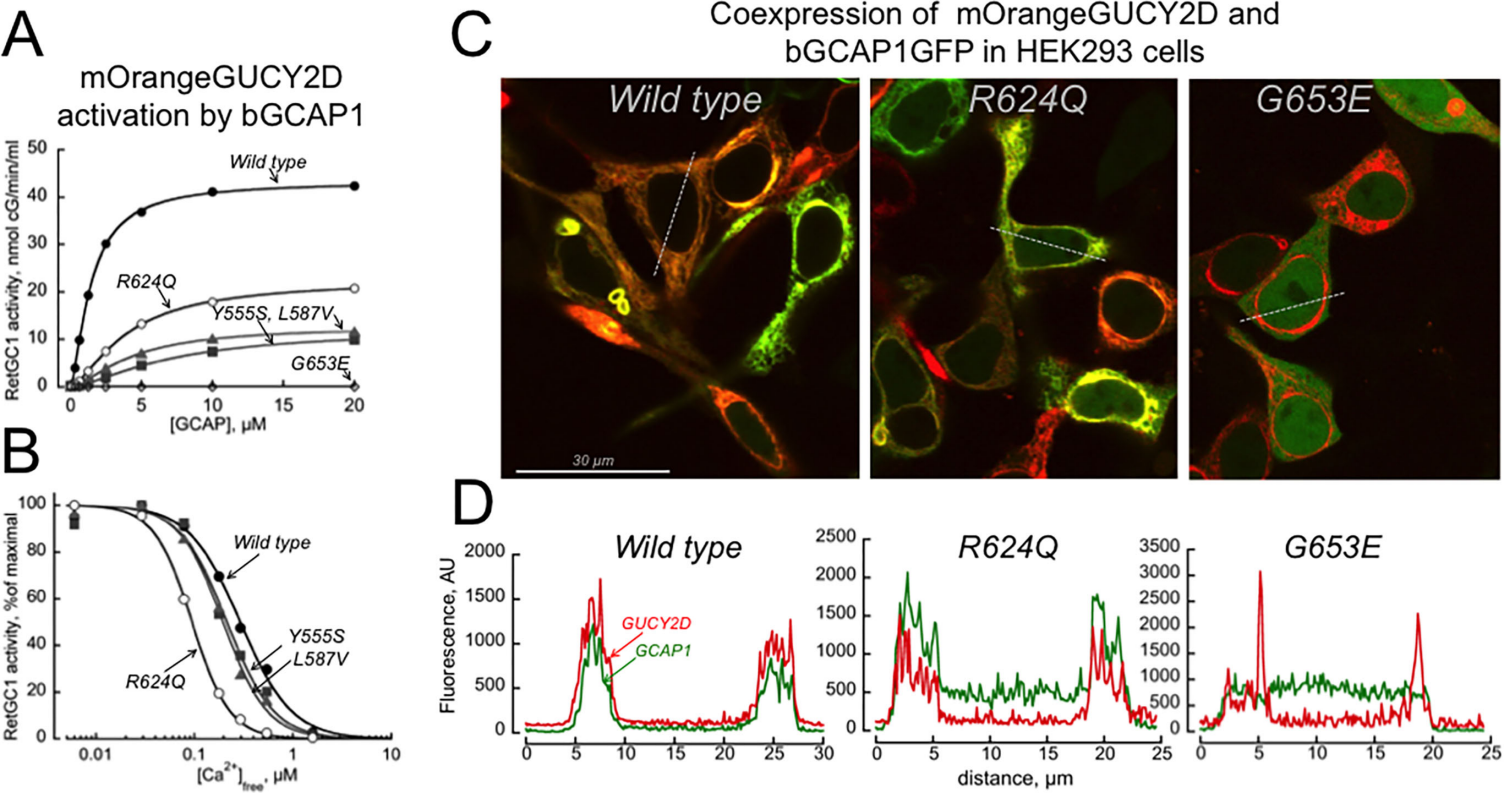
The effects of the *GUCY2D* mutations on the cyclase ability to bind GCAP1 in cyto. (**A**, **B**) Mutations found in SNB patients affect regulation of the mOrange-tagged *GUCY2D* cyclase activity similarly to the untagged *GUCY2D*, in both dose dependence of activation by GCAP1 (**A**) and Ca^2+^ sensitivity (**B**). mOrange*GUCY2D* (wild-type, Y555S, L587V, R624Q, or G653E) with the fluorescent tag replacing a portion of the extracellular domain of the cyclase was expressed in HEK293 cells with bovine GCAP1 (bGCAP1). (**C**) Confocal images of the mOrange–*GUCY2D* (red fluorescence) and bGCAP1GFP (green fluorescence) co-expressed in HEK293 cells. The HEK293 cells were co-transfected with the bGCAP1GFP (left to right: wild-type, R624Q, and G653E) and mOrange*GUCY2D* cDNAs at a molar ratio of ∼1:100 using FuGene reagent. (**D**) Fluorescence profile of the mOrange*GUCY2D* (red trace) and bGCAP1GFP (green trace) across the cells marked in panel **C**. Note the evidence of co-localization with GCAP1 in wild-type and R624Q *GUCY2D* and a complete lack of co-localization in G653E. The Y555S and L587V mOrange*GUCY2D* displayed co-localization patterns similar to that of the R624Q (not shown).

The activity of RetGC1 and its SNB mutants identified in this study was assayed in vitro in the presence of GCAP1 (Figs. 7^8^–9). We initially confirmed the presence of RetGC1 in the transfected cells by immunoblot and confirmed that the cells expressed similar levels of mutant RetGC1 variants and wild type (Fig. 7A). We then tested RetGC1 activation by GCAP1 and found that the four SNB mutants displayed either much lower than wild-type RetGC1 activity (Tyr555Ser, Leu587Val, and Arg624Gln) or no detectable activity at all (Gly653Glu), even at high concentrations of Mg^2+^ GCAP1 (Fig. 7B). Suppression of RetGC1 activity in the presence of high concentrations of GCAP2 and GCAP3 was also observed for all SNB mutants (Supplementary Fig. S2). This severe lack of activity was similar to that seen in null alleles associated with the *GUCY2D* LCA phenotype.^35^ The residual RetGC1 activity for the p.(Tyr555Ser) and p.(Leu587Val) mutants detectable in the presence of GCAP1 showed calcium sensitivity similar to that of the wild type, whereas calcium sensitivity for the p.(Arg624Gln) mutant was drastically elevated (Figs. 7C, 8, 9B). In that regard, Ca^2+^-sensitivity of the p.(Arg624Gln) mutant was shifted in the opposite way from that of the CORD6-linked variant, p.(Arg838Ser) (Fig. 8).^36^^,^^37^ This indicates that it would be even more difficult for GCAP to activate p.(Arg624Gln) RetGC1 when Ca^2+^ concentrations have declined after illumination, and this would even further restrain the already reduced enzymatic activity of the RetGC1/GCAP complex in rods and cones.

Another set of tests was conducted to evaluate the effects of *GUCY2D* mutations on the cyclase ability to bind GCAP1 in cyto, using co-localization of GFP-tagged GCAP1 with mOrange-tagged RetGC1 co-expressed in HEK293 cells (Fig. 9). When assayed in vitro, the mOrange-tagged RetGC1 and its mutants displayed biochemical characteristics of regulation by GCAP1 similar to those of the respective untagged cyclase variants (Fig. 9A vs. Fig. 7B), indicating the decreased affinity for GCAP1 in SNB mutants. Co-localization of RetGC1 mutants with GCAP1 observed in cyto using confocal imaging was consistent with the change in their apparent affinity for the activator protein observed in biochemical assays in vitro. Co-localization of the GFP-tagged GCAP1 and mOrange-tagged RetGC1 in the endoplasmic reticulum membranes was evident in the case of wild-type RetGC1, demonstrating a clearly defined co-distribution of both fluorochromes across the cells, but it was either poorly defined (Arg624Gln) or completely abolished (Gly653Glu) for the SNB mutant (Figs. 9C, 9D).

## Discussion

This study provides insights on the genetics and presentation of rare *GUCY2D*-associated night blindness based on detailed clinical, retinal imaging, and electrophysiological assessments. Distinctive structural and functional features were determined, including serial data that highlight a largely stable functional phenotype, at least until the fifth decade of life. Five previously unreported variants were identified, and functional testing revealed that four of these variants were not functionally different in vitro from those associated with *GUCY2D*-related LCA.

The phenotype of our patients largely matches that described by Rodilla et al.^11^ and Stunkel et al.^5^; however, some differences in our cohort are notable: (1) the retinal periphery in 10 of the 12 eyes showed temporal vascular attenuation, without bone-spicule–like pigmentation; and (2) all 12 eyes had abnormalities at the level of the foveal cone outer segments. Some of the patients described by the other groups were older than those presented here; hence, it is possible that pigmentary changes develop over time. Due to the retrospective nature of this clinical study, refraction data were not available. The combination of visual acuity of counting fingers or less and hypermetropia higher than +7 diopters has been reported as a feature of *GUCY2D*-related LCA. Characterizing the axial length and refractive error of SNB patients may provide insight into the role of RetGC1 in ocular development.^38^

The DA ERG phenotype was remarkably similar within our cohort of six patients with evidence of a complete or near-complete absence of rod photoreceptor function in all but one. Dim flash rod (DA 0.01) ERGs (selective for the rod system) were undetectable or residual in all but patient 2, and all six showed evidence that DA 10 (strong flash) ERGs were largely or entirely arising in the DA cone system; DA 10 ERG b-waves were of short peak time with similarities to cone-mediated DA red flash ERG x-waves and/or LA 3 ERGs, and some cases had a reduced DA 10 b:a ratio, likely representing cone-mediated activity (the “photopic hill” phenomenon manifesting under DA conditions in the absence of rod function).^36^^,^^37^ LA 30-Hz ERGs were delayed by 5 to 14 ms (median delay, 9.5 ms), and amplitudes were normal (*n* = 1), mild-moderately subnormal (*n* = 3), or severely subnormal (*n* = 2). There was PERG evidence of macular dysfunction in all but patient 3 (with a borderline/preserved P50 component). Patient 2 reported improved scotopic vision after extended periods in the dark, prompting further ERGs after overnight dark adaptation, but there was no ERG evidence of improved rod system function in the three patients tested. The same three patients had the mildest LA ERG reductions and underwent serial ERG testing over periods of 46 months (patient 2), 87 months (patient 3), or 157 months (patient 1), showing no evidence of worsening retinal or macular function in either eye. The ERGs and functional phenotypes in patients 1, 2, 3, and 6 are broadly consistent with those described in the six previously published SNB cases.^5^^,^^11^ A notable difference is that the previous reports described ERG worsening in two children(ages 12 and 9 years) who underwent repeat assessment at 17 and 15 years, respectively, including one with concomitant visual field constriction.^5^

All three patients whose color vision was tested using the HRR plates had a tritan defect, with patients 1 and 3 showing subnormal S-cone ERGs. Of the remaining three, only one was tested with Ishihara plates, which do not detect tritan defects. Tritan defects have been shown to be the earliest manifestation of *GUCY2D*-related CORD in asymptomatic patients, suggesting that S-cones are more vulnerable to abnormal RetGC1 function.^39^

Within our cohort, the alleles were located at the juxta-membrane, kinase homology, and cyclase catalytic domains of RetGC1, regions typically associated with null or very low/basal RetGC1 activity, due to an ineffective interaction with GCAPs.^9^^,^^10^^,^^40^ The variants p.(Arg768Trp), p.(Gly653Glu), and p.(Arg666Trp) were the previously reported alleles within our cohort. p.(Arg768Trp) is one of the most common *GUCY2D* LCA variants, and it is thought to represent a northwest European founder mutation.^41^^–^^43^ It affects a highly conserved residue and has been found to cause a severe reduction in *GUCY2D* activation by GCAP1; it has been reported in LCA patients in homozygous and compound heterozygous states.^9^ One individual with LCA was found to be a carrier of p.(Gly653Glu) (with no second allele found),^43^ and p.(Arg666Trp) has been reported in a patient with the night blindness phenotype in trans with a frameshifting allele.^5^

The variant p.(Leu587Val) has not been previously reported with this phenotype; however p.(Leu587Arg) has been reported to be associated with early-onset retinitis pigmentosa, without further details regarding zygosity and phenotype.^44^ p.(Tyr555Ser) and p.(Tyr909Cys) are also novel variants, with extremely low allele frequencies (Table 3). This mutation most likely directly affects the active site of the enzyme rather than its interaction with GCAP, because it is located in the catalytic domain, whereas GCAPs bind RetGC via the kinase homology domain and dimerization domains of RetGC1.^19^^,^^20^ The p.(Arg624Gln) has been reported in ClinVar as a Variant of Unknown Significance (Variation ID: 957183), associated with both LCA and CORD phenotypes, and it is present in population databases (rs746020263, counted five times, allele frequency of 0.00002%). This variant is in exon 12, outside of the mutation hot spot for CORD, and no segregation data were provided for the CORD case.^45^ Finally, p.(Gly653Gln) is absent from the literature and only appeared once in population databases (rs139168077, allele frequency of 0.000004%).

Our functional testing revealed that p.(Tyr555Ser), p.(Leu587Val), p.(Arg624Gln), and p.(Gly653Glu) disabled RetGC1–GCAP/retinal degeneration 3 (RD3) binding to various degrees, similar to LCA alleles.^35^ As the same alleles have been reported in LCA and night blindness, we cannot assume it is a particular location or conformational change that causes the different phenotypes. It is possible that the interaction between alleles partially neutralizes their effect, keeping a basal activity of RetGC1. If there was a residual rod function, the functioning cones might exert a strong lateral inhibition preventing recovery of rod function on ERG. However, overnight dark-adaptation failed to elicit measurable improvement of the rod function in our patients.

The role of RetGCs and their interactions with GCAPs and RD3 are of key importance in the phototransduction process; yet, the effects that these proteins have on each other have yet to be fully deciphered. In vitro and in vivo studies have shown that reducing the function of GCAP can leave RetGC without its activation at low Ca^2+^ concentrations in the light, creating cGMP production and restoring the permeability of CNG channels at basal levels only, causing a slower yet with larger amplitude response to light stimulus (resulting in loss of temporal resolution to bright stimuli).^46^^,^^47^ When RetGC is affected, the effect differs among variants associated with the CORD and LCA phenotypes. The former, located at the mutation hot spot of the dimerization domain (most frequently Arg838), have a dominant negative effect on RetGC1, decreasing its sensitivity to high intracellular calcium, thus causing an abnormal increase in cGMP production in the dark.^48^^,^^49^^,^^54^ LCA variants, on the other hand, prevent proper formation of the RetGC:GCAP complex or/and reduction of RetGC1 catalytic activity^32^^,^^35^ leading to reduced levels of cGMP, failure to open the proper fraction of the CNG channels, and a consequent reduction of the ERG a-wave.^8^ Rigg's type ERG is a rare form of congenital SNB in which constitutive activation of rod phototransduction leads to constant closure of the CNG channel and failure to produce graded potentials in response to photic stimulation.^36^ The ERGs reflect a functionally cone-isolated retina; however, in *GUCY2D*-related SNB, the cones are also partially dysfunctional, potentially suggesting the presence of a compensatory yet sluggish mechanism exclusive to the cones but not necessarily fully restoring their function.

Some of the difficulties in fully understanding these interactions are likely due to the lack of data regarding RetGC and RetGC–GCAP complex structure.^6^ Besides knowing RetGC1 location at the photoreceptor OS membrane,^50^ that rods have both RetGC1 and RetGC2 and GCAP1 and GCAP2, and that cones express almost solely GCAP1 and RetGC1,^51^^,^^55^ the nuances of such interactions have yet to be understood. Modeling this type of SNB using patient-derived retinal organoids could potentially contribute to our understanding of the pathophysiology of this condition. For example, it cannot be excluded^33^ that, in SNB patients, in contrast to LCA patients, cGMP regulation in L- and M-cones (less frequently in S-cones), but not in rods, for some reason becomes partially compensated by the second isozyme of RetGC, RetGC2 (GUCY2F), which usually presents a minor component in cGMP production.^26^^,^^52^ Why such compensation happens only in some individuals and results in the SNB phenotype but in more frequent cases it does not occur and results in LCA is difficult to explain at this time.

In conclusion, this study has detailed the largest cohort of patients with *GUCY2D*-associated SNB to date, to our knowledge. Variants leading to this rare presentation can affect multiple functional domains in *GUCY2D* and may also be associated with *GUCY2D* LCA. Patients often complain of experiencing night-vision issues since the late first decade of life, present mildly subnormal visual acuity and reduced color discrimination, and show subfoveal outer retinal abnormalities on OCT. Electroretinography may reveal a distinctive and relatively stable functional phenotype, characterized by severe loss of rod function with variable cone system involvement.

## Supplementary Material

Supplement 1

## References

[1] Liew G, Michaelides M, Bunce C. A comparison of the causes of blindness certifications in England and Wales in working age adults (16–64 years), 1999–2000 with 2009–2010. *BMJ Open*. 2014; 4(2): e004015. 10.1136/bmjopen-2013-004015 PMC3927710 24525390

[2] Glatz M, Riedl R, Glatz W, et al. Blindness and visual impairment in Central Europe. *PLoS One*. 2022; 17(1): e0261897. 35025896 10.1371/journal.pone.0261897 PMC8758103

[3] Kumaran N, Moore AT, Weleber RG, Michaelides M. Leber congenital amaurosis/early-onset severe retinal dystrophy: clinical features, molecular genetics and therapeutic interventions. *Br J Ophthalmol*. 2017; 101(9): 1147–1154. 28689169 10.1136/bjophthalmol-2016-309975 PMC5574398

[4] Kelsell RE, Gregory-Evans K, Payne AM, et al. Mutations in the retinal guanylate cyclase (RETGC-1) gene in dominant cone-rod dystrophy. *Hum Mol Genet*. 1998; 7(7): 1179–1184. 9618177 10.1093/hmg/7.7.1179

[5] Stunkel ML, Brodie SE, Cideciyan AV, et al. Expanded retinal disease spectrum associated with autosomal recessive mutations in GUCY2D. *Am J Ophthalmol*. 2018; 190: 58–68. 29559409 10.1016/j.ajo.2018.03.021

[6] Dizhoor AM, Peshenko IV. Regulation of retinal membrane guanylyl cyclase (RetGC) by negative calcium feedback and RD3 protein. *Pflügers Arch*. 2021; 473(9): 1393–1410. 33537894 10.1007/s00424-021-02523-4 PMC8329130

[7] Lamb TD, Pugh ENJ. Phototransduction, dark adaptation, and rhodopsin regeneration the proctor lecture. *Invest Ophthalmol Vis Sci*. 2006; 47(12): 5137–5152. 17122096 10.1167/iovs.06-0849

[8] Sharon D, Wimberg H, Kinarty Y, Koch KW. Genotype-functional-phenotype correlations in photoreceptor guanylate cyclase (GC-E) encoded by GUCY2D. *Prog Retin Eye Res*. 2018; 63: 69–91. 29061346 10.1016/j.preteyeres.2017.10.003

[9] Jacobson SG, Cideciyan AV, Peshenko IV, et al. Determining consequences of retinal membrane guanylyl cyclase (RetGC1) deficiency in human Leber congenital amaurosis en route to therapy: residual cone-photoreceptor vision correlates with biochemical properties of the mutants. *Hum Mol Genet*. 2013; 22(1): 168–183. 23035049 10.1093/hmg/dds421 PMC3606011

[10] Bouzia Z, Georgiou M, Hull S, et al. GUCY2D-associated Leber congenital amaurosis: a retrospective natural history study in preparation for trials of novel therapies. *Am J Ophthalmol*. 2020; 210: 59–70. 31704230 10.1016/j.ajo.2019.10.019 PMC7013380

[11] Rodilla C, Martín-Merida I, Blanco-Kelly F, et al. Comprehensive genotyping and phenotyping analysis of GUCY2D-associated rod- and cone-dominated dystrophies. *Am J Ophthalmol*. 2023; 254: 87–103. 37327959 10.1016/j.ajo.2023.05.015

[12] Ba-Abbad R, Holder GE, Robson AG, et al. Isolated rod dysfunction associated with a novel genotype of CNGB1. *Am J Ophthalmol Case Rep*. 2019; 14: 83–86. 30976726 10.1016/j.ajoc.2019.03.004 PMC6438912

[13] Turro E, Astle WJ, Megy K, et al. Whole-genome sequencing of patients with rare diseases in a national health system. *Nature*. 2020; 583(7814): 96–102. 32581362 10.1038/s41586-020-2434-2 PMC7610553

[14] Patel A, Hayward JD, Tailor V, et al. The oculome panel test: next-generation sequencing to diagnose a diverse range of genetic developmental eye disorders. *Ophthalmology*. 2019; 126(6): 888–907. 30653986 10.1016/j.ophtha.2018.12.050

[15] Robson AG, Frishman LJ, Grigg J, et al. ISCEV standard for full-field clinical electroretinography (2022 update). *Doc Ophthalmol*. 2022; 144(3): 165–177. 35511377 10.1007/s10633-022-09872-0 PMC9192408

[16] Thompson DA, Bach M, Sustar Habjan M, Viswanathan S, Robson AG (2024). ISCEV standard for clinical pattern electroretinography (2024 update). *Doc Ophthalmol*. 148(2): 75–85. 38488946 10.1007/s10633-024-09970-1 PMC10954931

[17] Sustar M, Holder GE, Kremers J, et al. ISCEV extended protocol for the photopic on–off ERG. *Doc Ophthalmol*. 2018; 136: 199–206. 29934802 10.1007/s10633-018-9645-y PMC6061123

[18] Perlman I, Kondo M, Chelva E, Robson AG, Holder GE. ISCEV extended protocol for the S-cone ERG. *Doc Ophthalmol*. 2020; 140: 95–101. 31749034 10.1007/s10633-019-09730-6

[19] Peshenko IV, Olshevskaya EV, Dizhoor AM. Dimerization domain of retinal membrane guanylyl cyclase 1 (RetGC1) is an essential part of guanylyl cyclase-activating protein (GCAP) binding interface. *J Biol Chem*. 2015; 290(32): 19584–19596. 26100624 10.1074/jbc.M115.661371 PMC4528125

[20] Peshenko IV, Olshevskaya EV, Dizhoor AM. Evaluating the role of retinal membrane guanylyl cyclase 1 (RetGC1) domains in binding guanylyl cyclase-activating proteins (GCAPs). *J Biol Chem*. 2015; 290(11): 6913–6924. 25616661 10.1074/jbc.M114.629642 PMC4358116

[21] Horton RM, Hunt HD, Ho SN, Pullen JK, Pease LR. Engineering hybrid genes without the use of restriction enzymes: gene splicing by overlap extension. *Gene*. 1989; 77(1): 61–68. 2744488 10.1016/0378-1119(89)90359-4

[22] Peshenko IV, Moiseyev GP, Olshevskaya EV, Dizhoor AM. Factors that determine Ca^2+^ sensitivity of photoreceptor guanylyl cyclase. Kinetic analysis of the interaction between the Ca^2+^-bound and the Ca^2+^-free guanylyl cyclase activating proteins (GCAPs) and recombinant photoreceptor guanylyl cyclase 1 (RetGC-1). *Biochemistry*. 2004; 43(43): 13796–13804. 15504042 10.1021/bi048943m

[23] Peshenko IV, Olshevskaya EV, Lim S, Ames JB, Dizhoor AM. Identification of target binding site in photoreceptor guanylyl cyclase-activating protein 1 (GCAP1). *J Biol Chem*. 2014; 289(14): 10140–10154. 24567338 10.1074/jbc.M113.540716 PMC3974984

[24] Peshenko IV, Olshevskaya EV, Dizhoor AM. Binding of guanylyl cyclase activating protein 1 (GCAP1) to retinal guanylyl cyclase (RetGC1). The role of individual EF-hands. *J Biol Chem*. 2008; 283(31): 21747–21757. 18541533 10.1074/jbc.M801899200 PMC2490776

[25] Olshevskaya EV, Boikov S, Ermilov A, Krylov D, Hurley JB, Dizhoor AM. Mapping functional domains of the guanylate cyclase regulator protein, GCAP-2. *J Biol Chem*. 1999; 274(16): 10823–10832. 10196158 10.1074/jbc.274.16.10823

[26] Peshenko IV, Olshevskaya EV, Savchenko AB, et al. Enzymatic properties and regulation of the native isozymes of retinal membrane guanylyl cyclase (RetGC) from mouse photoreceptors. *Biochemistry*. 2011; 50(25): 5590–5600. 21598940 10.1021/bi200491b PMC3127287

[27] Haeseleer F, Sokal I, Li N, et al. Molecular characterization of a third member of the guanylyl cyclase-activating protein subfamily. *J Biol Chem*. 1999; 274(10): 6526–6535. 10037746 10.1074/jbc.274.10.6526

[28] Peshenko IV, Dizhoor AM. Ca^2+^ and Mg^2+^ binding properties of GCAP-1. Evidence that Mg^2+^-bound form is the physiological activator of photoreceptor guanylyl cyclase. *J Biol Chem*. 2006; 281(33): 23830–23841. 16793776 10.1074/jbc.M600257200

[29] Mendez A, Burns ME, Sokal I, et al. Role of guanylate cyclase-activating proteins (GCAPs) in setting the flash sensitivity of rod photoreceptors. *Proc Natl Acad Sci USA*. 2001; 98(17): 9948–9953. 11493703 10.1073/pnas.171308998 PMC55558

[30] Peshenko IV, Yu Q, Lim S, Cudia D, Dizhoor AM, Ames JB. Retinal degeneration 3 (RD3) protein, a retinal guanylyl cyclase regulator, forms a monomeric and elongated four-helix bundle. *J Biol Chem*. 2019; 294(7): 2318–2328. 30559291 10.1074/jbc.RA118.006106 PMC6378972

[31] Peshenko IV, Dizhoor AM. Guanylyl cyclase-activating proteins (GCAPs) are Ca^2+^/Mg^2+^ sensors: implications for photoreceptor guanylyl cyclase (RetGC) regulation in mammalian photoreceptors. *J Biol Chem*. 2004; 279(17): 16903–16906. 14993224 10.1074/jbc.C400065200

[32] Peshenko IV, Olshevskaya EV, Dizhoor AM. Functional study and mapping sites for interaction with the target enzyme in retinal degeneration 3 (RD3) protein. *J Biol Chem*. 2016; 291(37): 19713–19723. 27471269 10.1074/jbc.M116.742288 PMC5016703

[33] Arden G, Gündüz K, Perry S. Color vision testing with a computer graphics system: preliminary results. *Doc Ophthalmol*. 1988; 69(2): 167–174. 3168720 10.1007/BF00153698

[34] Peshenko IV, Olshevskaya EV, Yao S, Ezzeldin HH, Pittler SJ, Dizhoor AM. Activation of retinal guanylyl cyclase RetGC1 by GCAP1: stoichiometry of binding and effect of new LCA-related mutations. *Biochemistry*. 2010; 49(4): 709–717. 20050595 10.1021/bi901495y PMC2827208

[35] Peshenko IV, Olshevskaya EV, Dizhoor AM. GUCY2D mutations in retinal guanylyl cyclase 1 provide biochemical reasons for dominant cone–rod dystrophy but not for stationary night blindness. *J Biol Chem*. 2020; 295(52): 18301–18315. 33109612 10.1074/jbc.RA120.015553 PMC7939455

[36] Zeitz C, Robson AG, Audo I. Congenital stationary night blindness: an analysis and update of genotype–phenotype correlations and pathogenic mechanisms. *Prog Retin Eye Res*. 2015; 45: 58–110. 25307992 10.1016/j.preteyeres.2014.09.001

[37] McCulloch DL, Kondo M, Hamilton R, et al. ISCEV extended protocol for the stimulus–response series for light-adapted full-field ERG. *Doc Ophthalmol*. 2019; 138: 205–215. 30929108 10.1007/s10633-019-09685-8

[38] Hanein S, Perrault I, Gerber S, et al. Leber congenital amaurosis: comprehensive survey of the genetic heterogeneity, refinement of the clinical definition, and genotype-phenotype correlations as a strategy for molecular diagnosis. *Hum Mutat*. 2004; 23(4): 306–317. 15024725 10.1002/humu.20010

[39] Hahn LC, Georgiou M, Almushattat H, et al. The natural history of Leber congenital amaurosis and cone–rod dystrophy associated with variants in the GUCY2D gene. *Ophthalmol Retina*. 2022; 6(8): 711–722. 35314386 10.1016/j.oret.2022.03.008

[40] Duda T, Venkataraman V, Goraczniak R, Lange C, Koch KW, Sharma RK. Functional consequences of a rod outer segment membrane guanylate cyclase (ROS-GC1) gene mutation linked with Leber's congenital amaurosis. *Biochemistry*. 1999; 38(2): 509–515. 9888789 10.1021/bi9824137

[41] Coppieters F, De Wilde B, Lefever S, et al. Massively parallel sequencing for early molecular diagnosis in Leber congenital amaurosis. *Genet Med*. 2012; 14(6): 576–585. 22261762 10.1038/gim.2011.51

[42] Yzer S, Leroy BP, De Baere E, et al. Microarray-based mutation detection and phenotypic characterization of patients with Leber congenital amaurosis. *Invest Ophthalmol Vis Sci*. 2006; 47(3): 1167–1176. 16505055 10.1167/iovs.05-0848

[43] Yohe S, Sivasankar M, Ghosh A, et al. Prevalence of mutations in inherited retinal diseases: a comparison between the United States and India. *Mol Genet Genomic Med*. 2020; 8(2): e1081. 31816670 10.1002/mgg3.1081 PMC7005662

[44] Avila-Fernandez A, Vallespin E, Cantalapiedra D, et al. Novel human pathological mutations. Gene symbol: GUCY2D. Disease: early onset retinitis pigmentosa. *Hum Genet*. 2007; 121(5): 650–651. 17879450

[45] Liu X, Fujinami K, Kuniyoshi K, et al. Clinical and genetic characteristics of 15 affected patients from 12 Japanese families with GUCY2D-associated retinal disorder. *Transl Vis Sci Technol*. 2020; 9(6): 2. 10.1167/tvst.9.6.2 PMC7408927 32821499

[46] Burns ME, Mendez A, Chen J, Baylor DA. Dynamics of cyclic GMP synthesis in retinal rods. *Neuron*. 2002; 36(1): 81–91. 12367508 10.1016/s0896-6273(02)00911-x

[47] Sakurai K, Chen J, Kefalov VJ. Role of guanylyl cyclase modulation in mouse cone phototransduction. *J Neurosci*. 2011; 31(22): 7991–8000. 21632921 10.1523/JNEUROSCI.6650-10.2011 PMC3124626

[48] Kitiratschky VBD, Wilke R, Renner AB, et al. Mutation analysis identifies GUCY2D as the major gene responsible for autosomal dominant progressive cone degeneration. *Invest Ophthalmol Vis Sci*. 2008; 49(11): 5015–5023. 18487367 10.1167/iovs.08-1901 PMC5358799

[49] Sato S, Peshenko IV, Olshevskaya EV, Kefalov VJ, Dizhoor AM. GUCY2D cone–rod dystrophy-6 is a “phototransduction disease” triggered by abnormal calcium feedback on retinal membrane guanylyl cyclase 1. *J Neurosci*. 2018; 38(12): 2990–3000. 29440533 10.1523/JNEUROSCI.2985-17.2018 PMC5864148

[50] Nemet I, Tian G, Imanishi Y. Organization of cGMP sensing structures on the rod photoreceptor outer segment plasma membrane. *Channels (Austin)*. 2014; 8(6): 528–535. 25616687 10.4161/19336950.2014.973776 PMC4594506

[51] Yang RB, Robinson SW, Xiong WH, Yau KW, Birch DG, Garbers DL. Disruption of a retinal guanylyl cyclase gene leads to cone-specific dystrophy and paradoxical rod behavior. *J Neurosci*. 1999; 19(14): 5889–5897. 10407028 10.1523/JNEUROSCI.19-14-05889.1999 PMC6783089

[52] Olshevskaya EV, Peshenko IV, Savchenko AB, Dizhoor AM. Retinal guanylyl cyclase isozyme 1 is the preferential in vivo target for constitutively active GCAP1 mutants causing congenital degeneration of photoreceptors. *J Neurosci*. 2012; 32(21): 7208–7217. 22623665 10.1523/JNEUROSCI.0976-12.2012 PMC3368705

[53] Laura RP, Dizhoor AM, Hurley JB. The membrane guanylyl cyclase, retinal guanylyl cyclase-1, is activated through its intracellular domain. *J Biol Chem*., 1996; 271(20): 11646–11651. 8662612 10.1074/jbc.271.20.11646

[54] Dizhoor AM, Olshevskaya EV, Peshenko IV. The R838S mutation in retinal guanylyl cyclase 1 (RetGC1) alters calcium sensitivity of cGMP synthesis in the retina and causes blindness in transgenic mice. *J Biol Chem*. 2016; 291(47): 24504–24516. 27703005 10.1074/jbc.M116.755553 PMC5114404

[55] Xu J, Morris L, Thapa A, et al. cGMP accumulation causes photoreceptor degeneration in CNG channel deficiency: evidence of cGMP cytotoxicity independently of enhanced CNG channel function. *J Neurosci*. 2013; 33(37): 14939–14948. 24027293 10.1523/JNEUROSCI.0909-13.2013 PMC3771030

