# Supplementary material for: Clinical and Biochemical Characterization of Specific GUCY2D Alleles Associated With a Rare Form of Night Blindness
Source: Invest Ophthalmol Vis Sci. 2025 Jun 6;66(6):22. doi: 10.1167/iovs.66.6.22 (PMC12155690; doi:10.1167/iovs.66.6.22)
Supplement: Supplement 1 [file iovs-66-6-22_s001.pdf]

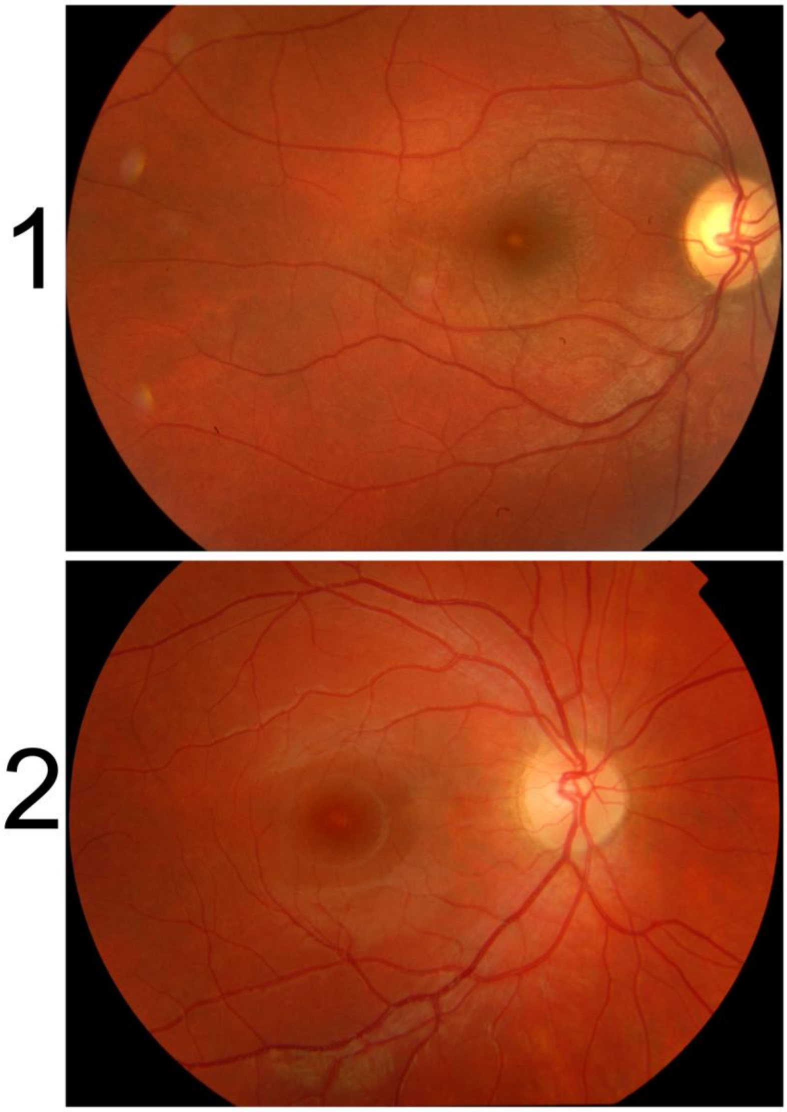

Figure S1: Macular color photographs of the right eye of patients 1 and 2. Note the distinct foveal hypopigmentation in both, which corresponds to the hyperautofluorescence in Figure 1.

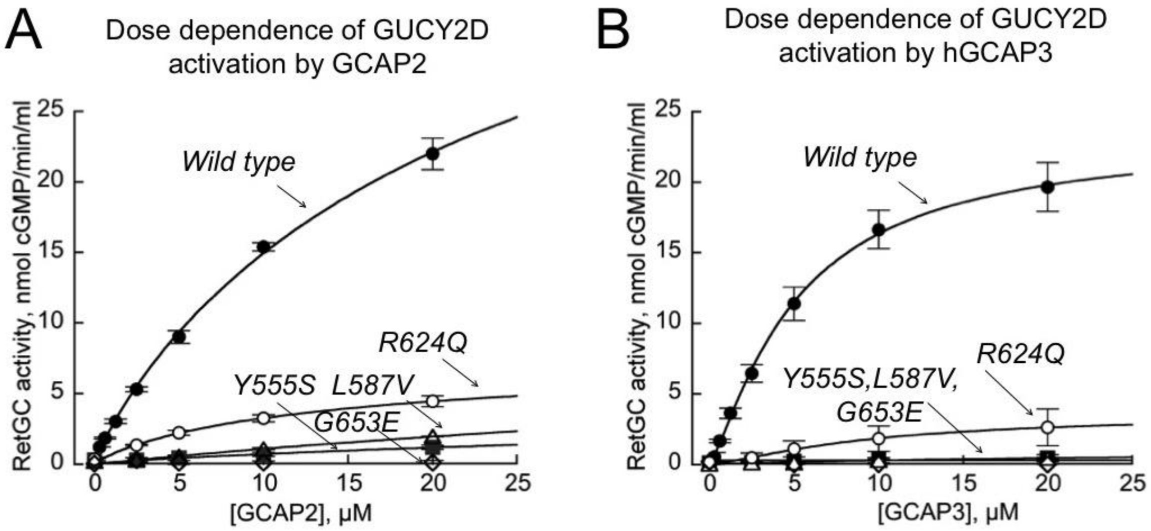

Figure S2: Dose-dependence of GUCY2D activation *in vitro* by recombinant human GCAP2 (A) and GCAP3 (B). The membrane fractions from HEK293 cells expressing wild type, Y555S, L587V, R624Q or G653E RetGC1 were reconstituted with purified recombinant human GCAPs and assayed at saturating 10 mM $MgCl_2$ in the presence of 2 mM EGTA as described in Figure 7B.
